# Objective Behavioral Measures in Children before, during, and after the COVID-19 Lockdown in Israel

**DOI:** 10.3390/ijerph18168732

**Published:** 2021-08-18

**Authors:** Einat Shneor, Ravid Doron, Jonathan Levine, Deena Rachel Zimmerman, Julia S. Benoit, Lisa A. Ostrin, Ariela Gordon-Shaag

**Affiliations:** 1 Department of Optometry, Hadassah Academic College, Jerusalem 9101001, Israel; eshneor@hac.ac.il (E.S.); ravidro@hac.ac.il (R.D.); jonathanle@edu.hac.ac.il (J.L.); 2 Maternal Child and Adolescent Division Public Health Services, Israel Ministry of Health, Jerusalem 9101002, Israel; deena.zimmerman@MOH.GOV.IL; 3 Texas Institute for Measurement, Evaluation, and Statistics, Houston, TX 77004, USA; Julia.Benoit@times.uh.edu; 4 College of Optometry, University of Houston, Houston, TX 77004, USA; lostrin@Central.UH.EDU

**Keywords:** COVID-19, pandemic, myopia, sleep, physical activity

## Abstract

Studies using questionnaires report that COVID-19 restrictions resulted in children spending significantly less time outdoors. This study used objective measures to assess the impact of pandemic-related restrictions on children’s behavior. A total of 19 healthy 8–12-year-old boys were observed before and during social restriction periods. Of these, 11 boys were reassessed after restrictions were lifted. For each session, Actiwatches were dispensed for measures of time outdoors, activity, and sleep. Changes overall and by school status were assessed using signed-rank test and Wilcoxon rank sum tests. During restrictions, children spent significantly less time outdoors (*p* = 0.001), were less active (*p* = 0.001), and spent less time engaged in moderate-to-vigorous physical activity (*p* = 0.004). Sleep duration was not significantly different between sessions (*p* > 0.99), but bedtime and wake time shifted to a later time during restrictions (*p* < 0.05 for both). Time outdoors and activity returned close to pre-pandemic levels after restrictions were lifted (*p* > 0.05 for both). Children’s behaviors significantly changed during the COVID-19 pandemic. The reduction in outdoor light exposure is of importance due to the role of light in the etiology of myopia and vitamin D production. The reduction in physical activity may have negative health effects in terms of obesity and depression, although further research is required to ascertain the long-term effects.

## 1. Introduction

Lifestyle factors, such as physical activity, outdoor light exposure, and sleep cycle have been shown to be important for children’s physical and mental health. Regular physical activity is recommended for children and adolescents to promote health and well-being [1]. Physical activity can help reduce risk factors for common noncommunicable diseases, including cardiovascular disease, some cancers, and depression [2]. Outdoor light exposure is important to protect children from vitamin D deficiency, which has been implicated in numerous diseases [3]. Furthermore, spending more time outdoors during childhood lowers the risk of developing myopia and may delay its progression [4]. Greater consistency in sleep timing may contribute to, or be reflective of, a healthier lifestyle, whereas greater bed-time variability is associated with a less healthy pattern of lifestyle behaviors [5].

The coronavirus disease 2019 (COVID-19) pandemic has had far-reaching health, social, and economic implications. Among them was the abrupt cessation of schooling for children and adolescents along with social distancing restrictions outside of school hours. Questions have been raised about how such COVID-19 restrictions might be affecting children’s physical and mental health. Numerous studies using questionnaires have measured the impact of social distancing and school closures on children’s physical activity, sleep, and screen time [6,7,8,9,10,11]. Most studies showed a decrease in physical activity [6,7,9,10,11] and increased screen time [6,7,8,9,10,11], but with equivocal results regarding sleep [6,7,9,10]. However, these outcomes are based on parent questionnaires, which are subjective and limited by recall errors and parental biases [12,13,14,15]. Another limitation of these studies is that they were based on cross-sectional surveys and did not directly measure pre-pandemic behaviors. Thus, they are measuring subjective perceived change. Some studies in adults have used objective longitudinal measures. In major metropolitan areas in different continents, smartphone app data have shown an increase in sleep during the pandemic [16]. A study using an accelerometer to measure physical activity showed a decrease in a small group of college students during the pandemic [17].

The aim of this study was to determine the impact of COVID-19 restrictions on children’s behaviors from objective measures of physical activity, time spent outdoors, and sleep parameters in a group of boys before and during the pandemic, and after pandemic-related social restrictions were lifted.

## 2. Materials and Methods

### 2.1. Participants

Before the pandemic, 36 boys participated in a study [18] with the goal of objectively assessing behaviors that are thought to contribute to myopia. Participants of that study were healthy boys aged 8.5–12 years, with best corrected visual acuity of 6/9 or better. Exclusion criteria were any anterior or posterior segment disease or pathology, such as strabismus and amblyopia, history of ocular trauma or surgery causing abnormal vision, and systemic diseases known to affect refractive error. During the pandemic, these same children and their families were contacted to see if they would participate in the current study, which included measurements during social restrictions and again after social restrictions were lifted. The current study was approved by the Ethics Committee of Hadassah Academic College and followed the tenets of the Declaration of Helsinki. The only inclusion criteria were having participated in the original study and a willingness to participate in this study.

**Figure 1:**
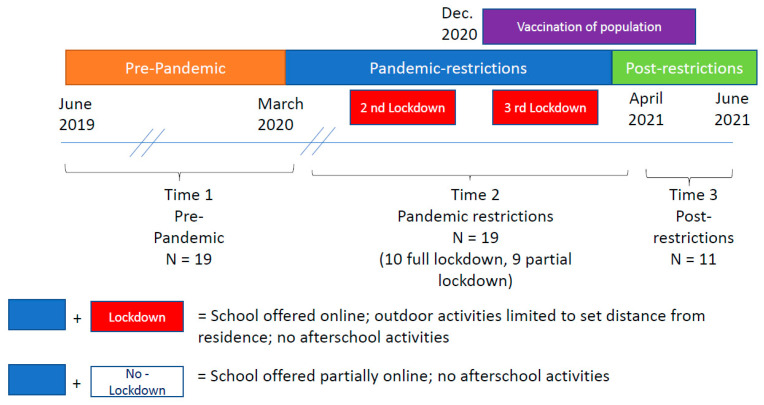
Timeline of Social Restrictions and Data Collection.

Thus, the current longitudinal study assessed the same group of boys in three time periods (Figure 1). Before the COVID-19 pandemic (time 1), during the pandemic when restrictions were in place (time 2) and after social restrictions were lifted (time 3).

### 2.2. Setting

From March 2020 to February 2021, the Israeli government declared three periods of full lockdown to lower the COVID-19 transmission rate. The first full lockdown was from 25 March 2020 to 3 May 2020, the 2nd from 18 September 2020 to 18 October 2020, and the 3rd from 27 December 2020 to 11 February 2021. During the first lockdown, individuals were required to stay within 100 m of their residence with the exception of using essential services [19]. During the 2nd, the area permitted was widened to 500 m [20] and during the 3rd it was widened to 1000 m [21]. During the full lockdowns, all regular school was conducted via online platforms. Between the full lockdowns, some physical classroom teaching was offered depending on the child’s grade and the number of active cases and transmission rate in the child’s neighborhood. When physical classroom teaching was offered, children were divided into small pods and each pod came to the school only a few days a week. Thus, during the pandemic, some of the children participated in this study when all schooling was online (full lockdown), and others participated when school was partially online and partially in the physical classroom (partial lockdown). Afterschool activities and youth groups did not meet during the entire period. On 21 December 2020, Israel started a campaign to vaccinate the entire population above age 16 years, resulting in a dramatic drop in new infections [22]. Starting in April 2021, school resumed to pre-pandemic status and after-school activities gradually resumed to pre-pandemic status. All restrictions within the country expired 1 June 2021. 

Pre-pandemic data were collected from June 2019 to March 2020. Data during social restrictions were collected from November 2020 until mid-March 2021. Post-social restrictions data were collected from April to June 2021 when school resumed returned to a normal schedule.

### 2.3. Measurements

The Actiwatch Spectrum (Philips Respironics, Bend, OR, USA) is a watch-like device that measures ambient light exposure and physical activity continuously at 32 Hz. The Actiwatch 2, a different model of the Actiwatch consisting of an accelerometer with similar range, bandwidth, and sensitivity as Actiwatch Spectrum used here, has been validated in children for physical activity [23] and sleep (duration, bed time, wake time) [24,25]. The Actiwatch has been actively used in both children and adults in various applications, including physical activity [26], sleep [27] and myopia-related studies [28,29,30,31,32]. The light sensor in the Actiwatch Spectrum consists of light sensitive photodiodes to measure illuminance of white light in units of lux. Physical activity is measured via a MEMS-type accelerometer and is expressed in counts per minute (cpm), a dimensionless measure of motion that is designed to remove the effects of gravity, transportation, and other types of acceleration that do not indicate subjects’ physical activity [33]. The accelerometer detects vertical accelerations between 0.5 and 2.0 g with a frequency response range of 0.35–7.5 Hz. The degree and speed of motion is integrated, the signal is amplified and digitized by the on-board circuit, and the data are stored in the memory of the device as activity counts. The device displays the time and date, and a sensor detects “off-wrist” time to monitor subject compliance. Each Actiwatch was dispensed for children to wear continuously for two weeks during the pre-pandemic and 10 days during the pandemic and post-restrictions and set to collect data at 1-min epochs. The epoch length was chosen to compare the results to previous studies in children using the Actiwatch Spectrum [18,34,35].

The wear time was shortened during the pandemic and post-restrictions periods to facilitate recruiting as many children as possible with a limited number of Actiwatches. Wear time was based on previous studies with accelerometers that found that a 1 week wear time provided reliable estimates of outdoor light exposure [36], and a 4 day [37] to one week [38] wear time was required for physical activity (including sleep) in adults. 

In this study, the Actiwatch was configured to average data over 1-min epochs, based on what is commonly used in accelerometry studies in children [26] and previous light studies in children [18,29]. Children were instructed not to remove the device for the entire measurement period and to take care not to cover the device with shirt sleeves or coats. Light exposure and activity counts were only included in the analysis when the subject wore the device for the entire day. Therefore, partial first and last days were excluded. Days were also excluded if the subject removed the device for more than 30 min, or if the light exposure dropped to zero for 30 min or more during daylight hours, indicating that the sensors on the device were obstructed by clothing. Only data from children who had at least 3 days of valid data were included in the analyses.

Actiwatch data were downloaded for each subject and included the following parameters per minute: off wrist, activity in cpm, white light (lux), interval status (active or sleep status). A day was defined from 12:00 a.m. to 11:59 p.m. Bedtime was defined as when the interval status changed from active to non-active. Wake time was defined as when the interval status changed from non-active to active. Time spent outdoors during daylight hours was defined as minutes exposed to >1000 lux. This cut-off point was based on the manufacturer’s recommendations, on a validation study in children [39], and from previous studies of light exposure and vision in children [18,28,29,30,32,36,40]. Total activity was calculated as mean daily cpm. Moderate-to-vigorous physical activity (MVPA) was defined as the number of minutes per day that each subject spent performing activity greater than 1048 cpm, based on a validation study of the Actiwatch and different types of physical activity in children [23] and previous research in children [30].

Daily duration of Jerusalem daylight was determined from timeanddate.com [41]. Mean daylight duration was assessed for potential differences between pre-pandemic, restriction, and post-restriction measurement periods.

### 2.4. Analysis

All measurements of time outdoors, physical activity, MVPA, sleep duration, bedtime, and wake time (pre-pandemic and during social restrictions) were analyzed univariately for outliers and proper distributional forms prior to statistical analysis. Approximate normality was assessed using quantile-quantile plots. All variables reported were summarized by means with standard deviations, medians with 25th and 75th percentiles, minimum, maximum or frequencies with percentages. Summary statistics are reported by COVID status (pre-pandemic versus during restrictions versus post-restriction) and compared using paired *t*-tests or signed-rank test for dependent comparisons, depending on the assumptions that can be made in each case.

To compare whether behavioral changes between pre-pandemic and pandemic differed by lockdown status (full versus partial), independent two-sample t-test or Wilcoxon rank sum test, were used where appropriate. To analyze differences in full 24-h activity profiles during pre-pandemic versus pandemic social restrictions, we first derived periods of time (using equal 4-h intervals) as a within-subject condition to indicate or represent relevant periods of time during the day. Intervals were defined as (1) sleep (12:00–3:59 a.m.), (2) early morning (4:00–7:59 a.m.), (3) school-time (8:00–11:59 a.m.), (4) school time (12:00–3:59 p.m.), (5) after school (4:00–7:59 p.m.), and (6) night time (8:00–11:59 p.m.). To determine whether patterns of change related to the pandemic differed by period of the day, a least square means (LSM) comparisons within a mixed model repeated measures approach was used, which flexibly handles correlated errors of two within-subject factors and utilizes maximum likelihood estimation. Using this approach, all observed responses from the six periods per 24 h and two measurement periods (pre-pandemic and pandemic social restrictions) were used. We included main effects period and measurement period (pre-pandemic vs. pandemic) and the period of day by measurement period interaction term. Of primary interest was the type 3 fixed effects, the hypothesis tests for the significance of each of the fixed effects, of the interaction term. If the interaction term was found to be statistically significant, post-hoc testing was performed to compare change in activity across period of the day and Tukey adjustments for multiple comparisons were applied.

A subset analysis was performed on boys who participated in the study for a third session, during the post-restrictions period. Friedman’s test, a non-parametric alternative to repeated measures ANOVA, followed by pairwise post-hoc test with Bonferroni correction, was used to compare behavioral changes between pre-pandemic, pandemic restrictions, and post-restrictions periods in this subset of boys. Statistical significance for all global tests was assessed at the 0.05 level. All statistical analyses were conducted using SAS 9.4, Stata 14.0 and SPSS 25.0.

Although the children wore the Actiwatches continuously for 24 h a day, the hours of daylight (sunrise to sunset) available when the children wore the watches was significantly shorter during social restrictions in comparison to pre-pandemic (10.9 ± 0.6 vs. 12.7 ± 1.5 h, *p* = 0.02) and was significantly longer post-restrictions than during social restrictions (13.6 ± 0.4 vs. 10.6 ± 0.5, *p* < 0.001). Since the available hours of daylight per day could impact outdoor light exposure, light exposure and activity were analyzed from 7:00 a.m. to 4:30 p.m., which were daylight hours during all three measurement sessions. This approach was used for all aggregate analyses.

## 3. Results

Of the 36 boys who participated in the experiment before the pandemic, 22 boys participated during the pandemic. The remaining 14 boys were either lost to follow-up (*n* = 5) or unable to schedule an appointment before school resumed post-restrictions (*n* = 9). Data from three boys during the pandemic were excluded: one boy found the watch uncomfortable so only wore it for one day, and two boys experienced watch malfunctions. Of the 19 boys included in the analyses, mean age pre-pandemic was 10.2 ± 0.9 years, and during the pandemic was 11.5 ± 0.9 years. Two pairs of boys (*n* = 4) were siblings. School was fully online when nine boys participated and partially online for 10 boys. An average of 15.3 ± 3.1 months passed between the pre-pandemic and pandemic time points (range 10.7–20.5 months). In total, 13 of the 19 boys wore the watch a third time after restrictions were lifted. One child experienced Actiwatch malfunctions and did not have enough data available to meet the inclusion criteria, and one child’s parents did not return the Actiwatch in a timely manner. The rest of the children (*n* = 6) were not able to participate in the limited amount of time between when restrictions were lifted and summer holiday, due to a limited number of Actiwatches. Thus, the analyses were performed on the data from eleven children. An average of 20.9 ± 2.7 months passed between the pre-pandemic and post-restrictions time points (range 15.2–23.3 months). 

When comparing behavior pre-pandemic and during social restrictions (Table 1), from 7:00 a.m. to 4:30 p.m., mean daily physical activity decreased from 595 ± 145 cpm to 429 ± 139 cpm (*p* = 0.001), moderate-to-vigorous physical activity decreased from 86 ± 39 min to 46 ± 30 min (*p* = 0.004), and daily time outdoors decreased from 1.8 ± 1.0 h to 0.7 ± 0.7 h (*p* = 0.001). Similar results were observed when all wake hours of day were included for physical activity (*p* < 0.03) and time outdoors (*p* < 0.01). 

Sleep duration was similar pre-pandemic (8.5 ± 0.7 h) and during pandemic social restrictions (8.4 ± 0.6 h, *p* > 0.99). However, boys went to sleep 0.97 ± 1.6 h later (*p* = 0.02) and woke up 1.1 ± 1.4 h later (*p* = 0.004) during social restrictions. 

When analyzing activity over a 24-h period, pattern differences between pre-pandemic and during social restrictions varied by time of day, as indicated by a statistically significant interaction between pandemic status and period (F = 17.4; *p* < 0.001). Table 2 shown a model that estimated least square mean differences (LSM) for physical activity. After adjusting for multiple comparisons, physical activity was significantly decreased during the pandemic restrictions relative to pre-pandemic for time periods reflecting school morning (8:00–11:59 a.m.: LSM: 267.4 cpm; *p* < 0.001); school afternoon (12:00 to 3:59 p.m.: LSM: 146 cpm; *p* < 0.001), and after school/evening (4:00 to 7:59 p.m.: LSM: 161.6 cpm, *p* < 0.001).

To assess whether different types of restrictions influenced the children’s behavior, data were further analyzed by the type of schooling children participated in during the pandemic. Nine children (47%) participated in the experiment when school was completely online (full lockdown), while 10 children (53%) participated when school was partially online and partially physically in the classroom (partial lockdown). In the pre-pandemic period, there was no statistically significant difference in physical activity between these two groups of children (full lockdown 610 ± 101 cpm (median: 635; 25th percentile 546; 75th percentile: 685) and partial lockdown 548 ± 158 cpm (median: 504; 25th percentile: 421; 75th percentile 674) *p* = 0.323). The magnitude of median reduction in average activity between the hours of 7:00 a.m. and 4:30 p.m. during the pandemic was larger among participants in full lockdown (−294 cpm (25th: −355, 75th: −219)) relative to participants in partial lockdown (−35 cpm (−113,−2)) (*p* = 0.002). Similar trends were found when analyzing time outdoors (Full: −93 (−145,−67); Partial: −30 (−67,−14); *p* = 0.008) and wake time (Full: 1.2 (0.6, 2.3); Partial: 0.6 (−0.2, 0.8), *p* = 0.047). Changes in sleep and bedtime were not found to differ across lockdown status (*p* > 0.05). These results suggest that not only were activity and time outdoors during the pandemic reduced compared to pre-pandemic, but that the magnitude of this reduction was larger among the most restricted group. 

**Figure 2:**
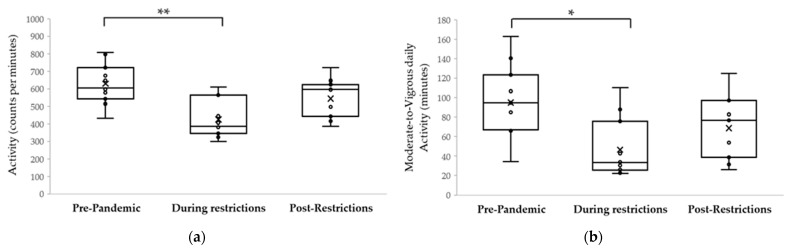

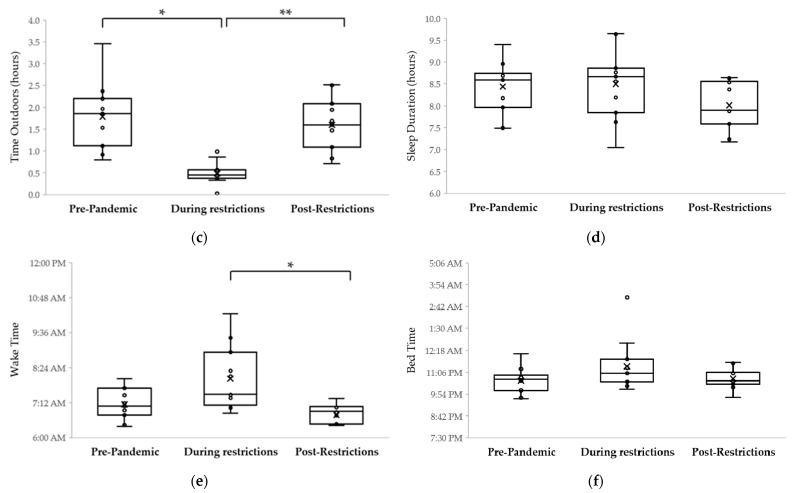
Plot illustrating different behaviors pre-pandemic, during restrictions and post-restrictions: (**a**) Mean daily physical activity (from 7:00 a.m. to 4:30 p.m.); (**b**) Mean moderate-to-vigorous daily physical activity (from 7:00 a.m. to 4:30 p.m.); (**c**) Mean daily time outdoors (from 7:00 a.m. to 4:30 p.m.); (**d**) Mean daily sleep duration (hours); (**e**) wake time; (**f**) bedtime. Pre-pandemic (black bars), during restrictions (gray bars), and post-restrictions (white bars) (*n* = 11); Solid horizontal line indicates the median, and the box extends between the 25th and 75th percentile of the data, and the whiskers extend to the smaller of the full range or 1.5 times the interquartile range of the data. * *p* < 0.05 and ** *p* < 0.001 for post-hoc pairwise test with Bonferroni correction between sessions.

Figure 2 shows physical activity, time outdoors, sleep, bedtime and wake time for the 11 boys who participated in the study on three occasions, pre-pandemic, during restrictions, and post-restrictions. Post-hoc analysis with Bonferroni correction found that, similar to the entire cohort, activity for these children was significantly reduced during restrictions (628 ± 116 cpm vs. 425 ± 107 cpm, *p* = 0.001). Post-restrictions, activity tended to increase (543 ± 110 cpm), but was not statistically different than during restrictions (*p* = 0.10). However, there was no statistically significant difference in activity between pre-pandemic and post-restriction (*p* = 0.10). In terms of time spent engaged in moderate-to-vigorous physical activity, these children spent less time in moderate-to-vigorous physical activity during restrictions (95 ± 37 min vs. 46 ± 30 min, *p* = 0.002). Post-restrictions, activity tended to increase (69 ± 32 min), but was not statistically different than during restrictions (*p* = 0.26).

On the other hand, sleep and time outdoors changed significantly post-restrictions compared to during restrictions (Friedman test: *p* = 0.04 and *p* = 0.001 respectively). Post-hoc testing with Bonferroni correction found that time outdoors was significantly reduced during restrictions (0.5 ± 0.3 h) compared to pre-pandemic (1.8 ± 0.8 h, *p* = 0.002), then returned to almost pre-pandemic levels (1.6 ± 0.6 h) after restrictions were lifted (post-restrictions vs. pandemic restrictions *p* = 0.001; post-restrictions vs. pre-pandemic *p* > 0.99). For sleep duration, after post-hoc with Bonferroni correction analysis, there were no significant differences pre-pandemic (8.4 ± 0.6 h) and during restrictions (8.5 ± 0.8 h, *p* > 0.99, Figure 2) and after restrictions were lifted (8.0 ± 0.6 h, *p* = 0.10). Similar to the entire cohort, wake time was shifted to a later hour during the pandemic social restrictions. The subset of boys tested post restrictions woke up significantly earlier once restrictions were lifted relative to during the pandemic (*p* = 0.004), with no overall effect of pandemic status period on bedtime (*p* = 0.34).

## 4. Discussion

This longitudinal study of children’s behavior using objective measures showed that children spent less time in outdoor light and were less active during periods when pandemic-related restrictions were in place compared to before the pandemic. The amount of time that children slept did not change during the pandemic, but bedtime and wake time shifted to be significantly later. The reduction in activity was at times of the day that correspond to when children would be in school and after school. The behavior of children in partial lockdown was less impacted than the behavior of children in full lockdown. Among those children who were also assessed after social restrictions were lifted, time spent in outdoor light and activity returned close to pre-pandemic levels. The current study is the first to provide objective longitudinal data regarding behavioral changes in children as a result of the COVID-19 pandemic.

Many questionnaires have assessed the impact of the pandemic on children’s physical activity and sleep. Self-report methods such as questionnaires, are characterized by their poor reliability and validity, especially in younger populations [14,15]. Thus, it is important to confirm questionnaires with objective measures. Questionnaires completed by parents regarding their children’s behavior have been shown to have biases [12]. Furthermore, there is often low agreement between primary and proxy respondents [42], even when the proxy respondent is a child’s parent [13]. 

### 4.1. Physical Activity

Behavioral changes observed during pandemic-related restrictions may have clinical consequences. Reduced physical activity is known to be associated with a negative impact on physical and mental health [43,44]. A systematic review found that physical activity interventions were associated with improved health outcomes for obesity, depression, high blood cholesterol, high blood pressure, the metabolic syndrome, low bone density, and injuries, and that the benefits were dose dependent for most conditions [2]. The correlation is that the reduced physical activity observed in the current study might have a negative impact of children’s health. The results of the current study provide objective support to the findings of prior studies using parent-reported questionnaires that investigated the impact of the pandemic in children around the world and found physical activity to be significantly reduced by social distancing restrictions [6,7,8,9,10,11,45,46]. Evidence has already been reported regarding negative short-term health outcomes of the pandemic. A review of 15 studies found that children gained weight and exhibited increased body mass index (BMI) in many places in the world during the COVID pandemic, due to both a reduction in physical activity and changes in diet [47]. There have been reports of increased pediatric mental health issues during the pandemic [6,48], and the reduced physical activity observed subjectively in questionnaires and objectively in this study may have a role.

The World Health Organizations recommends that children aged 5–17 years should do at least an average of 60 min per day of moderate-to-vigorous physical activity (MVPA) [1]. To determine if children met this daily goal, the metric measured in this study was minutes per day above 1048 cpm, which has been validated for the Actiwatch 2 against indirect calorimetry to correspond to MVPA [23]. Before the pandemic, the children in this study spent approximately 90 min per day engaged in MVPA, which was significantly reduced to 46 min during social restrictions. Children were meeting the daily requirement of physical activity before the pandemic, but not during social restrictions. In the subset for whom data are available post-restrictions, physical activity returned to above the daily recommendation of 60 min. Similar reductions in MVPA may contribute to the increased weight, BMI [47], and mental health issues [6,48] reported during the pandemic in some populations.

### 4.2. Time Outdoors

The reduction in time spent outdoors during the pandemic observed in this study might have clinical consequences for vitamin D deficiency and vision. Reduced time outdoors observed here may have resulted in Vitamin D deficiency, since the body received 80–90% of its Vitamin D from sun exposure. Thus, the results of this study raise red flags for children’s health. Vitamin D is important at all ages but even more so in children. Vitamin D plays a key role in calcium metabolism and thus bone growth which is at its peak during childhood [49]. Non-skeletal roles of Vitamin D continue to be discovered including an impact on the immune system in the prevention of both disease and autoimmunity [49]. A recent meta-analysis suggests that a normal level of Vitamin D has a positive influence on children’s mental health [50], which also may account for the mental health issues in children during the pandemic [6,48]. For these reasons many recommended oral Vitamin D supplementation during the pandemic [51].

Reduced time outdoors may also impact children’s eye growth and refractive error [52]. Accumulating evidence supports a role of outdoor light exposure in myopia (nearsightedness), with increased time outdoors being protective for myopia [52]. The findings of the current study using objective measures to quantify time outdoors support previous reports using subjective questionnaires, showing decreased time outdoors during the pandemic. Xu et al., studied myopia progression in children before and during the pandemic. Children’s outdoor activity and online schooling were assessed using a questionnaire [53]. The authors found that children’s outdoor time was negatively associated with increased myopia incidence and progression, and children’s online time was positively associated with increased myopia incidence. Furthermore, a recent study found that home confinement related to the pandemic was associated with a significant myopic shift for children ages 6 to 8 years [54].

### 4.3. Sleep

A reduction in sleep duration in children has been associated with [55], poor mental health [56] and obesity [57]. Greater bed-time variability is associated with a less healthy pattern of lifestyle behaviors [5]. The current study is the first to objectively measure sleep duration, wake time, and bedtime in children before and during pandemic-related restrictions. No differences in sleep duration were observed, however, bedtime and wake time significantly shifted later. Additionally, the range of these times was also greater during social restrictions. For example, before the pandemic, wake time ranged from 5:52 to 8:16 a.m., whereas during pandemic-related restrictions, wake time ranged from 6:39 a.m. to 12:04 p.m. Similarly, before the pandemic bedtime ranged from 9:06 p.m. to 12:38 a.m., and during pandemic-related restrictions, bedtime ranged from 9:21 p.m. to 4:37 a.m. Previous reports regarding the impact of the COVID-19 pandemic on children’s sleep using questionnaires are equivocal. Some studies found that sleep duration increased during the pandemic [6,7,9,10,46]. Nathan et al., found no difference in sleep duration [8], while Abid et al., found no difference in duration, but observed poorer sleep quality [45]. In an online survey, half of participants reported no difference in sleep duration during the pandemic, while a third reported increased sleep, and 17% reported decreased sleep [46].

### 4.4. School and Children’s Health

The results of this study highlight the impact of school closures on children’s health. In Israel, during the period of the pandemic when measurements sessions took place in this study, children were allowed to leave their homes for solitary (or with household members) physical activity. Thus, the reduction in physical active and time outdoors was not solely due to being restricted to their homes. This suggests that a regular school routine is essential for children to maintain healthy amounts of time outdoors and physical activity. The greatest reduction in physical activity was observed from 8:00 a.m. to 7:00 p.m. These are times of day that correspond to when children would have been at school and engaged in after-school activities, suggesting the importance of school for physical activity. The impact of the pandemic on children in partial and full lockdown supports this notion. The children who were in in-person school part time were significantly less disrupted by the pandemic in terms of physical activity and time outdoors than children who had no in-person school. Support for this notion comes from a systematic review on the impact of COVID-19 school closure on children’s health [58]. They found that school closures contributed to increased stress, sadness, and frustration. Furthermore, the longer the duration of school closure and reduction of physical activity, the higher the increase in BMI and prevalence of childhood obesity.

It should be noted that an innate difference in the behavior of the children in full and partial lockdown did not explain the different impact of the pandemic. On the contrary, before the pandemic the children in full lockdown were more active than their peers in partial lockdown, although this was not significant.

### 4.5. Post-Restriction Behavior

For the subset of boys who participated in post-restriction measures, time spent in outdoor light and activity returned to pre-pandemic levels once social distancing restriction were lifted and children returned to school full time. This finding further highlights the importance of a school routine for children’s normative behavior. Sleep duration did not change at any time point for these boys. However, they started waking up earlier after restrictions were lifted in comparison with pre-pandemic behavior. This might be due to the fact that they are in higher grades and school may start earlier. While behavior appears to have mostly returned to post-pandemic levels, it is not clear what the long-term health impact of the social distancing restrictions will be on children’s BMI, mental health, vitamin D levels and vision. These questions warrant longitudinal research. It would be important to assess these same children at future points in time to ascertain the continuity of their behavior.

### 4.6. Limitations

One of the limitations of this study is the small sample size; 19 children participated before and during the pandemic, and only 11 children participated after restrictions were lifted. However, this is the first longitudinal, objective study in children providing precise and continuous measures of physical activity, moderate-to-vigorous physical activity, light exposure, and sleep and is therefore valuable. Ideally, to make generalized conclusions regarding the impact of the pandemic on children’s behavior, we would have had to recruit a random sample of children before the pandemic, with the a priori plan of assessing them at three time points. Since we could not have predicted the pandemic and its subsequent social restrictions in advance, this was not possible. Although the results of this small sample should be interpreted with caution, we believe that this is valuable data and should be shared with the scientific community.

Since this is a small sample, it is not possible to represent the entire spectrum of a multicultural country. However, the children are from diverse Jewish ethnic backgrounds: nine were religious, five were secular and five were ultra-Orthodox, as classified by their school system. Furthermore, they represented a spectrum of socioeconomic strata (based on their cities of residence). All children in this study were originally recruited before the pandemic by word of mouth and advertisements posted at the Hadassah Academic College Eye clinic, which has a diverse patient base since it provides subsidized eye care regardless of socioeconomic background. A previous study did not find differences in physical activity and outdoor light exposure between religious, secular and ultra-Orthodox boys [18]. Furthermore, a questionnaire of Israeli Arab children found similar results regarding physical activity and sleep during the pandemic [6]. As restrictions were uniformly applied and enforced throughout the country, it is unlikely that behavior would be diverse between different ethnic groups. Similarly, in a small sample, one cannot study a large age range of children. All the children included in this study were ages 8–12 years before the pandemic; thus, the findings may not reflect pre-school or high school children’s behaviors.

This study only included boys because it is a follow up of a previous study that only included boys. In terms of physical activity and sleep, some studies evaluating physical activity and sleep during the pandemic using questionnaires reported no significant differences between boys and girls in terms of the impact of the pandemic [6,7,8,10], although one did report a difference in physical activity [46]. Thus, it is hard to predict what the results would have been had we included girls.

Another limitation of this study is that the children are all in Israel and may not represent children in other parts of the world. However, the results of this objective study confirm questionnaire studies on children from around the world, including Italy and Spain [9,10], China [11,46], Australia [8], Canada [7], and Tunisia [45]. Thus, it is likely that findings in the current study may be generalizable to those in other part of the world who underwent pandemic-related restrictions.

Another limitation of this study is the difference in seasons between the three time points. Data were collected before the pandemic from June 2019 to March 2020. Pandemic restrictions during the first lockdown and the subsequent summer and Jewish school Holiday breaks, led to data being collected during the pandemic-related restrictions from November 2020 to March 2021. Thus, the data were collected in different seasons, and there was a significant difference in average available daylight time, which we controlled for by limiting analysis of time outdoors and activity to only 7:00 a.m. to 4:30 p.m., times that had daylight at every time point in the study. Furthermore, previous studies found that outdoor light exposure did not vary by season to the magnitude found in the current study. A study in adults in Australia did not find a significant difference in light exposure between winter and summer for myopic children and only a half hour difference for emmetropic children [59]. A study of children the in the United States found a seasonal difference in daily outdoor light exposure in the range of 20–30 min [29], which is much smaller than the change found in the current study. Thus, it is likely that much of reduction in light exposure was due to social distancing restrictions and not available light exposure. In terms of sleep duration, a previous study with Actiwatches found that a small seasonal difference of 11.5 min [35], unlike the current study which did not find a significant difference in sleep duration. Similarly, we hypothesize that the reduced physical activity observed in the current study during pandemic restrictions was not due to an effect of season. As mentioned above, the study in the United States found an effect of season on time outdoors; however, physical activity was not significantly different between seasons [29]. On the other hand, children in Norway [60,61], Portugal [62], and England [63] were more active in warmer, drier seasons, and children in Australia were more active in winter than summer [64,65]. Interestingly, in the latter study, boys did not exhibit seasonal fluctuation in physical activity [64]. The weather in Israel is more similar to Australia than to Norway or England. Therefore, if there were seasonal variations, we might expect boys to be more active in fall and winter. Our findings show that boys were least active during fall and winter when social restrictions were in place.

During the pandemic, children were assessed in two distinct periods—some in full lockdown and some in partial lockdown. Both periods demonstrated significant changes in children’s behavior in comparison to pre-pandemic period. Different strategies of social distancing used in different countries may impact generalizability of the findings. However, the fact that this study described children’s behavior during two different strategies of social distancing (full lockdown and partial lockdown) makes the findings more applicable to other countries.

Lastly, there are currently few validations studies of the Actiwatch Spectrum and none in children. The parameters for wear time and epoch length used in this study were based on validation studies using with either the Actiwatch 2 [24,25], or where it was not clearly stated which Actiwatch model was used [23]. The cutoff defined for moderate to vigorous physical was based on a validation study of physical activity that used 15 s epochs [23], in contrast to the 60 s used in the current study. Since children’s physical activity is composed of short bouts, this may have missed some short-duration vigorous activity. Despite these shortcomings, the same parameters were used in this study as in previous studies of light exposure, physical activity, and sleep using the same device (Actiwatch Spectrum) [18,34,35], so as to compare the results. Furthermore, the main finding was the change in behavior at different points of time for the same subject. Thus, the absolute magnitude is less important than the comparison between time points. 

## 5. Conclusions

The objectively measured findings in this study carried out in Israel, together with previous questionnaire-based studies from all over the world, show that when school schedules were impacted due to the pandemic, children were less physically active, spent less time in moderate-to-vigorous physical activity, spent less time outdoors, and had altered bedtimes and wake times. Interventions should focus on encouraging children to actively play outside within the confines of the social restrictions of a given setting with a focus on activities that provide moderate-to-vigorous physical activity. Additionally, clinicians should be aware of physical and mental health issues that may arise as sequelae from pandemic-related behavioral changes. It would be important to assess these same children in the future to ascertain the continuity of their behavior.

## Figures and Tables

**Table 1:** Mean and range of daily physical activity and time outdoors (from 7:00 a.m. to 4:30 p.m.), sleep duration, and bed and wake times pre-pandemic and during social restrictions (*n* = 19).

	Pre-Pandemic ^β^	Social Restrictions ^β^	*p* Value *
Physical activity (counts per minute)	595 ± 145	429 ± 139	0.001 ^£^
	596 (459, 719)	385 (345, 563)	
	(353–806)	(168–736)	
Time MVPA (minutes per day)	86 ± 39	46 ± 30	0.004
	85 (49, 123)	33 (25, 76)	
	(27–163)	(1–110)	
Time outdoors (hours per day)	1.8 ± 1.0	0.7 ± 0.7	0.001
	1.6 (1.1, 2.2)	0.5 (0.4, 0.8)	
	(0.3–4.4)	(0.0–3.0)	
Sleep duration (hours per night)	8.4 ± 0.6	8.5 ± 0.7	>0.99
	8.6 (7.6, 8.9)	8.7 (8.2, 8.9)	
	(7.4–9.4)	(7.0–9.7)	
Bedtime	10:35 p.m. ± 53 min	11:34 p.m. ± 109 min	0.02
	10:42 p.m. (9:42 p.m.–11:17 p.m.)	11:13 (10:20 p.m., 12:03 a.m.)	
	(9:06 p.m.–12:06 a.m.)	(9:21 p.m.–4:38 a.m.)	
Waketime	7:04 a.m. ± 38 min	08:12 a.m. ± 89 min	0.004
	7:05 a.m. (6:36 a.m., 7:33 a.m.)	8:04 a.m. (7:01 a.m.–8:55 a.m.)	
	(5:53 a.m.–08:16 a.m.)	(6:39 a.m.–12:05 p.m.)	

* *p* value < 0.05 represents significant differences between sessions; ^β^ Descriptive Statistics are reported as: Mean ± SD; Median (25th percentile, 75th percentile); and Range (minimum—maximum); ^£^ Indicates parametric test was used; Abbreviations: MVPA—moderate-to-vigorous physical activity; min—minutes.

**Table 2:** Estimated change in activity during social restrictions compared to pre-pandemic by 4-h intervals within a 24-h period (*n* = 19).

Estimated Difference in Activity (Pre-PANDEMIC MINUS during Restrictions)	Estimated Mean Change (cpm)	95% CI	*p* Value
12:00 to 3:59 a.m.	14	−34, 62	>0.99
4:00 to 7:59 a.m.	−62	−111, −13	0.33
8:00 to 11:59 a.m.	−267	−348, −186	<0.001
12:00 to 3:59 p.m.	−146	−227, −65	<0.001
4:00 to 7:59 p.m.	−162	−243, −81	<0.001
8:00 to 11:59 p.m.	−27	−108, 54	>0.99

## Data Availability

The data presented in this study are available on request from the corresponding author.
